# Study of the influence of elemental composition variability in MoCrN coatings on stability and wear resistance

**DOI:** 10.1039/d5ra04839g

**Published:** 2025-11-10

**Authors:** Ye. A. Kenzhin, D. I. Shlimas, A. M. Zikirina, A. L. Kozlovskiy, V. V. Uglov

**Affiliations:** a Kazakh-British Technical University Almaty Kazakhstan; b NAS Eurasian National University ofL.N. Gumilyov Astana Kazakhstan kozlovskiy.a@inp.kz; c Belarusian State University Pr. Nezavisimosti 4 Minsk 220050 Belarus

## Abstract

The paper presents the assessment results of the applicability of MoCrN coatings as wear-resistant protective coatings capable of withstanding extreme operating conditions associated with both exposure to aggressive environments and high temperatures typical for their use as thermal barrier anti-corrosion coatings for the protection of steel structures. The coatings were obtained using the magnetron sputtering method, by variation of the power of which high-strength coatings with different ratios of elements in the composition, the change of which ensures variability of resistance to external influences and corrosion processes, are obtained. During the tribological tests of the studied coatings, it was found that the displacement of chromium from the composition of MoCrN coatings due to its substitution by molybdenum results in enhancement of resistance to destruction and wear, as well as an increase in the stability of the coating surface as a result of tribo-corrosion tests associated with the simultaneous impact of an aggressive environment and a rubbing body. According to the obtained results of changes in the friction coefficient of the studied samples, it was established that a change in the elemental composition of the coatings leads to a more than 2–5-fold increase in wear resistance, both in the case of standard tribological tests and after tribocorrosion tests. Enhancement of the stability of coatings by alteration of the elemental composition by substitution of chromium by molybdenum leads to a growth in resistance to corrosion processes by more than 1.5–2 times, which indicates a positive effect of variation of the composition of coatings and an increase in their resistance to external mechanical influences.

## Abstract 2

The paper presents the assessment results of the applicability of MoCrN coatings as wear-resistant protective coatings.

## Graphical abstract

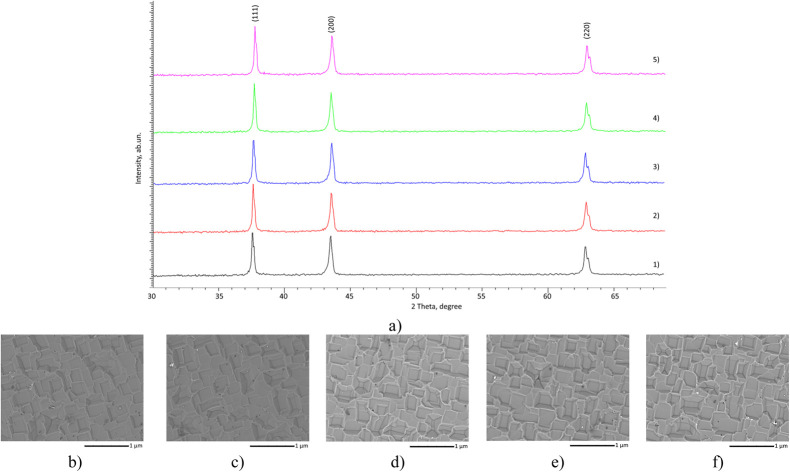

## Introduction

The increase in demand for wear-resistant materials used in extreme conditions is primarily due to the development of industry, as well as the need to reduce production costs with the constant rise in prices for fuel and metals.^1,2^ One of the ways to solve the problem of reducing production costs is the use of wear-resistant protective or so-called “sacrificial” coatings, which ensure an increase in the service life of the main cutting elements or structures used in extreme conditions, including aggressive environments, high temperatures or prolonged mechanical friction.^3–5^ The technology of their application is based on the mechanism of creating a protective “buffer” layer on the surface of steel structures with a thickness from several hundred nanometers to tens of microns, which will serve as a buffer between the surface of the steel and aggressive environments, as well as mechanical impacts exerted on the structures during their operation. In this case, much attention is paid not only to the possibility of protection from the negative consequences of external influences, but also to the possibility of reducing friction and the rate of degradation due to buffer protective coatings or layers.^6,7^ In most cases, various oxide, oxy-nitride, nitride or carbide coatings, as well as various polymer or silicon-containing coatings applied to the surface of steel structures using various methods, are considered as protective coatings. In this case, the choice of the applied coating is usually based on the expected conditions of its operation, including exposure to aggressive environments, high temperatures, mechanical loads, *etc.*^8–10^

As is known, variation in the ratio of components in the composition of coatings due to the effects of alloying with metals leads to the formation of a sliding effect, which in turn leads to a decrease in the coefficient of friction and an increase in resistance to wear in dry or vacuum environments. However, in most cases, the effectiveness of resistance decreases when using such coatings in conditions of exposure to aggressive environments, which is accompanied by tribooxidation processes associated with constant contact of the coating with an aggressive environment under the influence of friction.^11^ In turn, the use of coatings with the addition of graphite or diamond-like carbon, which have high hardness and wear resistance under long-term exposure, due to the formation of a transfer layer supersaturated with carbon, as well as surface graphitization processes, also limits the possibilities of using them in aggressive environments, due to their high sensitivity to interaction with oxygen or aggressive environments. Also, one of the key problems of carbon-containing coatings is their low adhesion to the substrate surface, which does not allow obtaining coatings of large thickness, which also limits the service life of the coatings due to their destruction and peeling at small thicknesses. The use of nitride coatings that combine several elements, such as titanium, chromium, molybdenum or aluminum, allows for increased resistance to tribooxidation processes that occur in aggressive environments under mechanical impact, due to structural features, as well as a sufficiently dense packing of layers, which allows for slowing down oxidation processes.^12–16^ At the same time, the combination of substitution effects when two types of elements, for example, molybdenum and chromium, aluminum and chromium, are used, allows for increased resistance to external influences, as well as to destruction processes caused by exposure to aggressive environments due to the possibility of combination of the structural features of the elements used in the composition of the coatings. For example, partial substitution of some elements by others contributes to a change in structural features, which in turn leads to an increase in resistance to deformation distortions, as well as tribooxidation processes, accompanied by the formation of oxide films or abrasive at the point of contact of the rubbing body with the surface.^17–19^ High resistance of coatings to external mechanical impacts, together with their strength and heat-insulating properties, makes it possible to increase the service life of structural steels and alloys significantly. However, despite the great prospects for using coatings as protective materials, there are still many unresolved issues related to both the methods of their production and the selection of optimal compositions with the best indicators of protection from external impacts.^20–22^

The aim of this work is to determine the prospects for using MoCrN coatings obtained by variation of the conditions of magnetron sputtering, the changes of which lead to variability in the elemental composition of the coatings, as protective wear-resistant and thermal barrier coatings that prevent the degradation of steel structures. The hypothesis of this work is based on the assumption that variation in the ratio of elements in the composition of coatings will increase resistance to external influences, as well as increase wear resistance under mechanical influences. The main reason for the use of this type of coating is the ability to combine the high strength and wear resistance of MoN coatings with the high resistance to corrosion processes when exposed to aggressive environments or high temperatures of CrN coatings. Moreover, in contrast to the works that consider layer-by-layer alternation of layers, in this work the method of simultaneous deposition, which made it possible to create a ternary system Mo(Cr)N, the formation of which occurs due to the partial substitution of molybdenum with chromium in the structure of the cubic lattice of the MoN phase, was used. The combined presence of molybdenum and chromium in the nitride matrix promotes the formation of thermodynamically stable solid solutions and also allows for the regulation of tribological characteristics, increasing resistance to external influences during operation in high-temperature conditions. Thus, MoCrN is a promising coating for use in conditions of intense wear and aggressive environments, as well as in case of high-temperature exposure.

## Materials and methods

The coatings were produced using magnetron sputtering on an Auto 500 (Edwards) setup. Molybdenum and chromium targets were used as targets for producing the coatings. The targets were 99.95% pure and were manufactured by K. Lesker, USA. A 35/65 mixture of argon and nitrogen was used as the working gas, with a gas pressure of approximately 5 × 10^−3^ bar. The coatings were applied using the high-frequency magnetron sputtering method, with the sputtering power range from 200 to 300 W. The power supplied to the cathodes was the same, which, due to differences in sputtering coefficients and atomic binding energies at variation of the power, leads to a controlled change in the Mo/Cr ratio and the phase formation of a stable structure during reactive nitriding. Before opening the shutter, the targets were pre-sputtered in Ar (without N_2_) for 10–15 min with the shutter closed to clean the surface of the targets. The N_2_ content of 65% allowed for the formation of a stable nitride phase with a cubic type of crystal lattice, while regulation of the spray power variation allowed for the elemental ratio of components in the coating composition to be varied (see data in Table 1).

**Table 1:** Elemental composition and structural parameters of MoCrN coatings

Parameter	Sputtering power, W				
	200 W	220 W	250 W	270 W	300 W
Ratio of elements Mo/Cr/N, at%	23/33/44	26/32/42	27/31/42	34/25/41	41/19/40
Crystal lattice parameter, Å	*a* = 4.1412 ± 0.0015	*a* = 4.1362 ± 0.0018	*a* = 4.1329 ± 0.0017	*a* = 4.1313 ± 0.0013	*a* = 4.1238 ± 0.0021

The substrates used for coating application were 316L steel and aluminum nitride, the choice of which was due to the possibility of assessment of the effect of coatings on enhancement of wear resistance under tribological influence, as well as testing the coating resistance to hot corrosion processes under the influence of an open flame. In this case, to test the samples for resistance to hot corrosion, coatings were applied to aluminum nitride ceramics, which have high resistance to thermally induced embrittlement and creep, characteristic of 316L steel. The substrate samples were prepared by grinding and polishing to achieve a roughness of 5–10 nm. The sample dimensions were 3 × 3 cm^2^.

The possibility of sputtering power variation in the range from 200 to 300 W made it possible to make changes in the ratios of elements during sputtering, as a result of which it was determined that an increase in power leads to the dominance of molybdenum in the composition, the change in concentration of which is from 23 to 41 at% in the case of an increase in power from 200 to 300 W at maintenance of almost stable weight contribution of nitrogen in the composition of the coatings (see data in Table 1).

**Fig. 1:**
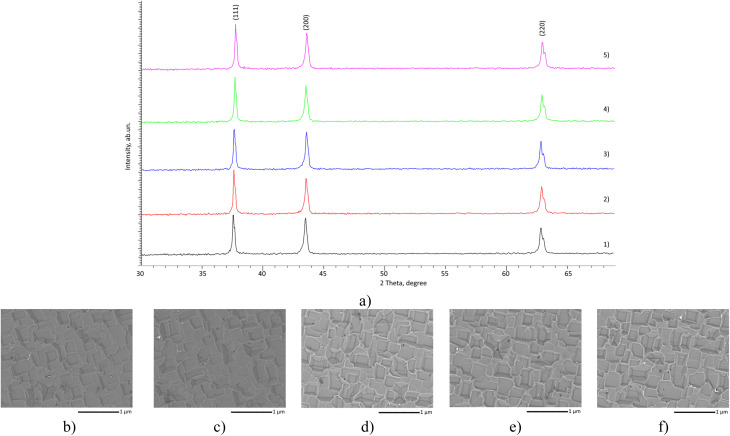
(a) Results of X-ray diffraction of the studied MoCrN coatings, obtained for samples at sputtering power variation: (1) 200 W; (2) 220 W; (3) 250 W; (4) 270 W; (5) 300 W; SEM images of the sample surface, reflecting the morphological features of the obtained coatings depending on the sputtering power: (b) 200 W; (c) 220 W; (d) 250 W; (e) 270 W; (f) 300 W.

Elemental analysis data were obtained using the energy-dispersive analysis method implemented on a TM3030 scanning electron microscope (Hitachi, Tokyo, Japan). Also, using this microscope, the images of the coating surface were obtained, shown in Fig. 1.

In this case, the change in the elemental composition of the coatings is due to the fact that when the magnetron sputtering power grows from 200 W to 300 W, the key effect during sputtering is the differences in the sputtering coefficients and the binding energies of the atoms. When the power is increased, not only does the plasma density grow but so does the energy of ion bombardment, which leads to increased ionization processes and re-scattering of Cr atoms, which have a lower mass and a higher secondary desorption coefficient. At the same time, Mo atoms, which are characterized by a higher mass and lower volatility, are retained in the flow and deposited on the substrate, thereby forming a stable cubic phase of MoN. As a result, a decrease in chromium content and a predominance of molybdenum in the coating composition are observed. Thus, variation of the magnetron sputtering power is a key factor controlling the balance between the elements and determining the final coating stoichiometry.

The analysis of structural parameters and phase composition of the studied MoCrN coatings depending on the conditions of their sputtering was carried out using the X-ray diffraction method. The implementation of this method was carried out using a D8 ADVANCE ECO diffractometer (Bruker, Karlsruhe, Germany), the diffraction patterns were recorded using the Bragg–Brentano method, in the angular range of 2*θ* = 30–70°, with a step of 0.05°. The obtained diffraction patterns presented in Fig. 1 were not subjected to smoothing, nor to subtraction of the kα2 function, and the observed shift of reflections on the diffraction patterns is primarily associated with the results of substitution caused by the processes of variation of the conditions of coating synthesis. According to the diffraction patterns presented in Fig. 1a, the assessment of the phase composition of the obtained coatings made it possible to determine that the phase composition of the coatings is represented by the cubic phase MoN (PDF-01-076-3075) with spatial syngony *Fm*-3*m* (225), as well as a fairly good structural ordering degree (more than 85%). The assessment of the structural parameters presented in Table 1 confirms the observed shift of reflections to the region of large angles, associated with the difference in the ionic radii of Cr – 0.073 nm and Mo – 0.062 nm. An increase in the molybdenum concentration in the coating composition leads to a reduction in the crystal lattice distortions and a decrease in the Mo(Cr)–N type crystalline bond distortions, due to a decline in the contribution of cationic substitution in the octahedra.

Fig. 1b–f demonstrates the results of the morphological features of the obtained coatings depending on the variation of the sputtering conditions (power changes). As can be seen from the data presented, the coating microstructure consists of cubic or diamond-shaped grains, forming fairly densely packed grains with clearly defined boundaries. The observed microstructural features of the coatings, when the component ratios vary, do not show significant differences when considered in general. This effect is due to the fact that the resulting coatings have the same phase composition, and the main changes are associated with variations in the components in the composition and a decrease in the parameters of the crystal lattice associated with substitution effects. In the case of samples obtained at 280–300 W, according to the scanning electron images, the presence of grains of various shapes is present, but their concentration is insignificant.

Coating hardness was determined using microindentation using a Duroline M1 microhardness tester (Metkon, Bursa, Turkey). The measurement mode was adjusted so that the indenter's impression on the sample would correspond to the coating without a substrate. Low loads of approximately 10 N were used for this purpose. A Vickers diamond pyramid was used as a pyramid; the indenter's exposure time on the surface was 15 seconds, after which the geometry of the imprint and the dimensions of the diagonals were determined. Based on this the hardness of the coating was determined. The number of measurements for each sample was approximately 20, with the average distance between measurements being more than 30–50 μm to avoid the interference of deformations caused by indentation with the hardness determination in the subsequent measurement.

The adhesive strength of the coatings was assessed using the scratch test method, which allows determining the maximum pressure that must be applied to remove the coating from the substrate surface. The tests were carried out using a UNITEST 750 testing machine (Ducom Instruments, Bengaluru, India). The adhesive strength was determined by application of a variable load to the indenter, the change of which results in separation from the substrate. Based on the scratch tests, the load values at which the coating separates from the substrate were determined, and their analysis made it possible to evaluate the effect of variation in the ratio of the component elements in the coating composition on the resistance to separation.

Tribological experiments were carried out using a T100 tribometer, equipped with the ability to conduct experiments both in standard mode when determining the coefficient of dry friction, and in the case of heating samples and exposure to aggressive environments, by conducting experiments in special cups designed for tribo-corrosion testing. SiC balls with a diameter of 10 mm were used as counterweights, and the tests were conducted using the reciprocating motion method (ASTM G133). New balls were used for each measurement. The path length was 2 cm in both directions, and the total path length was 400 m (the number of cycles was 20 000). The speed was 0.2 m s^−1^, and the frequency was 5 Hz. The maximum pressure generated by the ball was approximately 1.2–1.5 GPa at a ball load of 10 N, and the maximum shear stress was approximately 0.3–0.4 GPa.

The effect of variations in the composition of MoCrN coatings on tribological characteristics was assessed by determining the friction coefficient under various conditions, including dry friction, as well as in the case of surface heating and simultaneous exposure to an aggressive environment. The experiments were conducted to identify the role of changes in the ratio of components in the coating composition on wear resistance during wear resistance tests, as well as to assess the applicability of the proposed coatings as materials capable of operating under extreme conditions. Wear resistance tests were conducted in three different experiments. The first series of experiments was implemented under standard conditions for determining the coefficient of dry friction at a constant load on the ball equal to 10 N, the number of cycles of action (repetitions 20 000 cycles), a silicon carbide ball was used as an indenter (counterbody). The assessment of the surface heating at the point of contact between the counterbody and the surface during friction was carried out by measuring the temperature using a thermal imager and thermocouples. The second series of tests included an assessment of the effect of heating on coating samples with the simultaneous effect of an indenter during tribological tests with a ball load of 100 N, heating was carried out by placing the samples on a special platform heated to a temperature of 500 °C, heating was controlled using thermocouples fixed on both sides of the samples, allowing one to assess the temperature on the surface of the sample subjected to mechanical loads. The third series of tests included the effect of an acidic environment (0.1 M HCl) on the surface of the coatings and their role in the loss of wear resistance under simultaneous exposure to an aggressive environment and mechanical stress. The tests were carried out with an indenter load of about 100 N, the number of test cycles was 20 000 cyclic repetitions, which made it possible to compare the results of the experiments with each other, as well as to identify the role of additional external influences on the wear resistance and resistance of coatings to destruction processes.

The applicability of the proposed MoCrN coating compositions as protective materials for thermal degradation associated with dynamic oxidation under the influence of an open flame was assessed by conducting experimental tests by heating samples to temperatures of about 1300 °C and holding them at this temperature for a specified time of about 100 hours. This experiment aims to evaluate changes in the processes of direct flame exposure from a torch to the surface of coating samples, allowing for the assessment of dynamic oxidation of the coating surface during prolonged exposure to an open flame. The selection of these conditions allowed simulation of more aggressive operation, which differs from uniform isothermal heating in a furnace.

To conduct these experiments, a gas burner with a TORGWIN collet cylinder, the application of which allows achievement of a maximum temperature in the main torch zone of about 1300 °C, was used. To maintain constant exposure of the open flame to the surface of the coatings, the cylinders were changed every 5–10 hours. After holding the coating samples under the specified conditions, the coating hardness, adhesive strength and wear resistance were assessed using tribological tests. In this case, the tests were carried out using a torch flame by direct exposure of the flame to the surface of the coating, the thickness of which was about 2 μm and the area of the sample was 3 × 3 cm^2^, which made it possible to concentrate the flame on the central part of the sample. In this case, the samples were applied to the surface of aluminum nitride, which is resistant to thermally induced creep processes characteristic of 316L steel, the use of which limits the possibility of conducting experiments assessing dynamic oxidation when exposed to an open flame.

## Results and discussion

As a rule, wear-resistant coatings are used to protect against corrosion, leading to the destruction of near-surface layers, as well as to increase the service life of materials, especially those subject to mechanical stress when in contact with aggressive environments or thermal heating. At the same time, one of the key objectives in this area is the possibility of enhancement of resistance to mechanical impacts and corrosion processes that occur when exposed to high temperatures or aggressive environments with mechanical friction, which leads to accelerated surface wear processes.

**Fig. 2:**
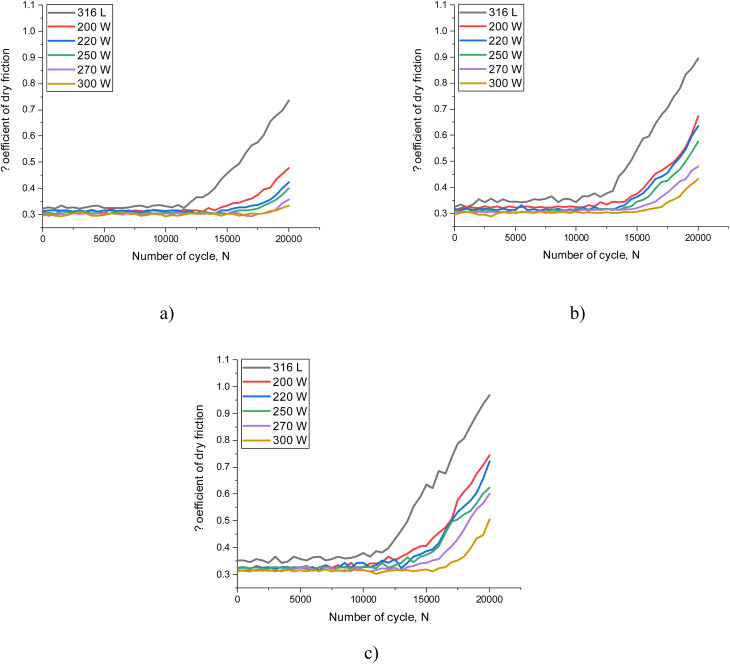
Results of the evaluation of tribological characteristics (dry friction coefficient) depending on the variation of experimental conditions: (a) in case of a standard test scheme; (b) in the case of thermo-tribological tests with simultaneous heating of samples and mechanical friction; (c) in the case of tribo-corrosion experiments under the simultaneous influence of an aggressive acidic environment and mechanical friction.

Fig. 2 reveals the results of tribological tests of MoCrN coatings in the case of different experimental schemes, described in detail in the “Materials and methods” section. To compare the influence of the efficiency of the applied coatings on the resistance to mechanical wear during long-term mechanical action on the samples, tribological tests of 316L steel, used as a substrate on which the coatings were applied, were carried out in all experiments. The general view of the changes in the dependence of the friction coefficient on the number of cycles is characterized by two sections with different trends of changes: the incubation section, characterized by small changes in the friction coefficient, indicating the stability of the surface and good adhesion of the counterbody to the surface; the transition section, in which a growth in the friction coefficient value, caused by the instability of the surface associated with its partial destruction and increased wear, is observed. The results of the evaluation of the wear resistance of 316L steel, given for all three types of tests, indicate a fairly strong wear of the steel surface and an increase in the friction coefficient after 10 000–12 000 test cycles, while the observed changes in trends depending on the type of tribological experiments indicate an acceleration of the wear process, as well as more pronounced destruction, both in the case of thermo-tribological tests and tribo-corrosion tests for wear resistance. The growth of the degradation trend, especially in the case of thermal exposure and tribo-corrosion experiments, indicates the acceleration of the processes of destruction of the steel surface associated with the formation of oxide inclusions, the presence of which leads to the acceleration of surface destabilization. In this case, during thermal heating, the acceleration of destruction processes is caused by oxidative wear, which is associated with the formation of an oxide film on the surface of the steel, the formation of which leads to peeling and the creation of additional barriers for the counterbody, which leads to an increase in the friction coefficient. In this case, the appearance of oxides in the near-surface layer also leads to a loss of strength and an increase in wear, which is clearly demonstrated by the increase in the dry friction coefficient value, as well as the trend of its changes depending on the number of test cycles. In the case of tribo-corrosion tests in an aggressive environment, the simultaneous impact of an aggressive environment and mechanical friction on the steel surface leads to the destruction of the passive film that protects against the aggressive environment, and the resulting pitting accelerates the destruction process due to the growth of deformation stresses and distortions, the accumulation of which leads to cracking and increased wear. In this case, surface degradation leads to an increase in plastic deformation due to the accumulation of pits in the structure, an increase in the proportion of which leads to an increase in friction and, consequently, a loss of wear resistance. At the same time, comparing the results of the change trends, it can be concluded that in the case of 316L steel, surface degradation is most pronounced when exposed to an aggressive environment, which is associated with the acceleration of corrosion processes due to the constant impact of the counterbody during friction on the contact surface with the aggressive environment, which leads to acceleration of destruction due to the disruption of the passive layer and the formation of corrosion products. At simultaneous impact of an aggressive environment and a counterbody during tribo-corrosion tests, the observed acceleration of the destruction processes of the near-surface layer is due to the acceleration of the surface corrosion processes associated with the penetration of oxygen, chlorine and hydrogen ions deep into the samples through microcracks formed as a result of mechanical action. In this case, the penetration of ions into the depth leads to destabilization of the crystalline structure due to the formation of deformation distortions, as well as the filling of micropores, which subsequently serve as nuclei for pitting corrosion. Acceleration of the damaged layer destruction processes results in loss of adhesion of the near-surface layer, which leads to its peeling, and consequently, growth of the abrasive, leading to a rise in resistance of the counterbody during tribological tests. In this case, the observed rapid growth in the friction coefficient for steel indicates not only acceleration of wear, but also loss of surface strength as a result of the simultaneous impact of an aggressive environment and mechanical friction.

In the case of tribological tests with applied MoCrN coatings under standard conditions (the first series of experiments described in the Materials and methods section), it is evident that the coefficient values, regardless of the ratio of elements in the composition, are of the order of 0.295–0.310, which is somewhat less than the values established for 316L steel, for which the value was of the order of 0.320–0.325. Such small differences in values are due to the morphological features of the obtained coatings, the cellular structure of which leads to an insignificant decrease in friction. It should also be noted that the observed decrease in the friction coefficient with an increase in the concentration of molybdenum in the coating composition can be explained by the effects of creating a passivating film on the surface, reducing friction under mechanical action. During comparison of the results of the trends of changes in the dependences of the friction coefficient on the number of cycles obtained for coatings and 316L steel, it can be concluded that for coatings the incubation period, which characterizes the preservation of surface stability and low wear, is about 15 500–16 000 cycles in the case of standard friction conditions, and about 13 000–14 000 cycles in the case of thermo-tribological and tribo-corrosion tests. In this case, for 316L steel, the incubation period is about 10 000–12 000 cycles, which is 15–20% less compared to the results presented for coatings. It is important to highlight that during assessment of the change in surface temperature during tribological tests, in the case of 316L steel, the surface heating after 10 000 cycles at the point of contact of the counterbody and the surface is about 70–100 °C, while with the use of protective coatings, the heating is about 40–50 °C, which is approximately 1.5–2 times lower than for steel samples subjected to similar tests. According to the data obtained, the occurrence of the effect of thermal heating of the surface of samples as a result of mechanical impacts (during friction) in the case of prolonged exposure can lead to the acceleration of the processes of surface destabilization associated with the occurrence of microcracks, the growth of which reduces stability and leads to peeling of the surface, which in turn increases the friction coefficient due to the formation of an abrasive at the point of interaction.

By analyzing the observed changes in the coefficient of dry friction and their relationship with structural changes caused by variations in the ratio of elements in the composition of the coatings, it can be concluded that the observed compaction of the crystal lattice leads to an increase in adhesion, which in turn leads to a decrease in wear. At the same time, the observed deformation distortion at high concentrations of chromium in the coating composition under prolonged mechanical action contributes to the acceleration of wear, which is clearly seen in the trends of the dependence of changes in the dry friction coefficient for samples obtained with a sputtering power of 200–250 W, for which the observed growth in the dry friction coefficient value after 15 000 cycles significantly exceeds similar trends obtained for samples with a sputtering power of 270–300 W, for which molybdenum dominates in the coating composition.

In the case of thermo-tribological and tribo-corrosion tests of MoCrN coatings, the observed changes in trends in comparison with the standard scheme for conducting tribological tests indicate a negative influence of external factors on the stability of the coatings, however, it should be noted that the observed trends and the nature of the changes are much less intense than in the case of similar tests of the surface of 316L steel.

In turn, the increase in resistance to external influences during mechanical friction for coatings with a high molybdenum content can be explained by the effect of the formation of a viscous MoO_3_ layer, the formation of which allows the counterbody to “float” in the contact zone with the coating surface, thereby reducing friction, as evidenced by a reduction in the friction coefficient value, and, as a consequence, less pronounced destruction of the surface during long-term tests. A similar effect was discovered in a number of studies, according to which the formation of a viscous MoO_3_ film on the surface of the coating during mechanical tests leads to a decrease in the friction coefficient and an increase in wear resistance.^23,24^ Moreover, such an effect is manifested not only in the case of standard conditions of mechanical impacts, but also in the case of high-temperature impacts, which in turn leads to an increase in the resistance of coatings to wear. At the same time, in the case of thermal heating, at a temperature much higher than the temperature required to initiate the oxidation processes of molybdenum, the formation of a viscous-flowing layer of molybdenum oxide occurs more intensively, which initially leads to a slowdown in wear, but in the case of prolonged exposure to an acceleration of the destruction processes, leading to an increase in the proportion of abrasive consisting of flakes exfoliated from the near-surface layer, which results in destabilization of the surface and an increase in friction resistance (*i.e.* an increase in the friction coefficient depending on the number of test cycles). During tribo-corrosion tests, the acceleration of destruction processes due to the penetration of corrosion products deep into the near-surface layer of coatings results in acceleration of the loss of wear resistance, however, a change in the ratio of components in the composition of MoCrN coatings leads to an increase in wear resistance under the simultaneous influence of an aggressive environment and mechanical friction.

**Fig. 3:**
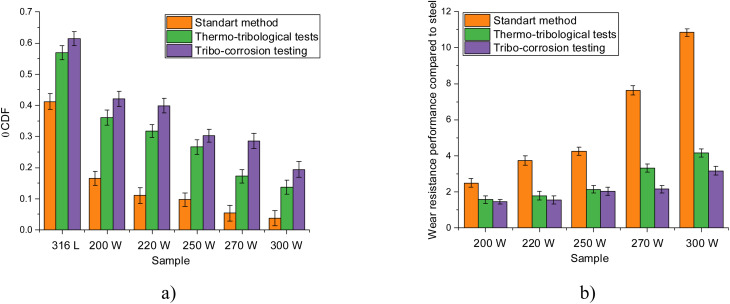
(a) Results of the assessment of changes in the friction coefficient for MoCrN coatings depending on the conditions of tribological experiments; (b) results of wear resistance evaluation of MoCrN coatings in comparison with the results obtained for 316L steel.

Based on the obtained values of the friction coefficient at the beginning of the tests and upon reaching 20 000 cycles, the change parameters reflecting the degree of destruction and wear of the surface were determined. The results are presented in Fig. 3a. Fig. 3b illustrates the comparison results of the values of the efficiency of alteration of the wear resistance of the applied coatings in comparison with the results obtained for 316L steel.

As is evident from the presented data on the assessment of the change in the friction coefficient (ΔCDF) after various types of testing, the greatest difference in values is observed for 316L steel, for which the difference in ΔCDF in the case of the standard scheme is 0.4, and with thermo-tribological and tribo-corrosion tests, the growth in the friction coefficient value after the end of the tests is about 0.57–0.61, which is 1.5 times higher than the values of ΔCDF obtained for the standard scheme. The application of coatings to the surface of 316L steel results in a significant reduction in wear resistance and an increase in resistance from 2.2 to 11 times compared to the results of changes in the ΔCDF value obtained for 316L steel (see data in Fig. 3b). In the case of thermo-tribological and thermo-corrosion tests, the wear resistance in comparison with the results obtained for steel 316L is significantly lower than in the case of the standard test scheme, however, the obtained values of efficiency and wear resistance indicate a positive effect of using the proposed coatings to increase the resistance to degradation of 316L steel. It should also be mentioned that the greatest efficiency in the case of thermo-tribological and tribo-corrosion tests is achieved for coating samples in which molybdenum predominates. For them, the efficiency of wear resistance is about 3.5–4 times, which is more than 2 times greater than similar values obtained for coatings with a high chromium content in the composition.

The obtained assessment results of the efficiency of enhancement of resistance to wear during mechanical friction in the case of additional external influences (heating or aggressive environment) indicate the potential of using such coatings to protect steel from corrosion processes associated with combined external influences, the acceleration of which results in rapid destabilization of the near-surface layers. Moreover, the possibility of stability enhancement by application of coatings to the surface allows for a reduction in the destruction rate, which in turn increases the service life and, as a result, reduces the cost of replacement of corroded parts during operation in extreme conditions.

Protective coatings play an important role in protecting metal structures not only from mechanical impact processes (friction, scratching, pressure), but also from high-temperature corrosion, which occurs both in the case of metal contact with heating elements and in the case of exposure to open flame, contact with which is accompanied not only by the formation of oxide films, but also by melting processes in the case of direct long-term exposure to metal.

**Fig. 4:**
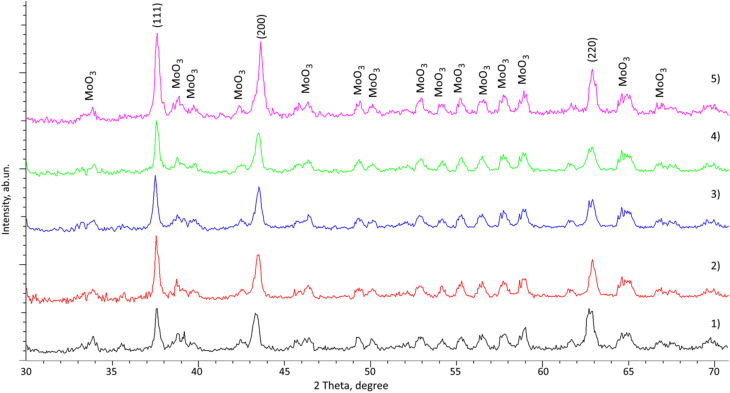
Results of X-ray phase analysis of the studied MoCrN coatings after hot corrosion tests: (1) 200 W; (2) 220 W; (3) 250 W; (4) 270 W; (5) 300 W.

Fig. 4 shows the results of X-ray diffraction of MoCrN coating samples after hot corrosion tests, reflecting the phase changes in the samples after testing. The main changes observed in the X-ray diffraction patterns, in comparison with the results shown in Fig. 1a, are associated with both a change in the intensities of diffraction reflections, indicating structural disordering processes, and the formation of impurity inclusions characterized by the appearance of a large number of strongly broadened low-intensity reflections of asymmetric shape. An analysis of the phase composition of the reflections carried out to identify these data made it possible to establish that the observed reflections correspond to a highly distorted orthorhombic phase of MoO_3_, the appearance of which in this case may be associated with the processes of oxidation and the formation of this phase in the form of individual inclusions with a highly disordered structure, as evidenced by the shape of the diffraction maxima, which has a strong asymmetry, as well as a broadened shape. As is known, the formation of the oxide phase, as a rule, occurs at temperatures above 400 °C, as a result of the introduction of oxygen into the crystalline structure with the subsequent displacement of the impurity phase in the form of metastable inclusions. The presence of deformation distortions of the crystal lattice, as well as interstitial defects, leads to the facilitation of the diffusion of oxygen deep into the near-surface layer, which leads to the acceleration of surface destabilization with the possibility of the formation of impurity inclusions in the near-surface layer. It should be noted that variation in the ratio of components in the composition of the coatings leads to a less pronounced formation of the oxide phase, as evidenced by the change in the ratio of weight contributions obtained by analysis of the areas of reflections on diffraction patterns (see data in Table 2). The observed change in the ratio of weight contributions in the composition of the coatings with variations in the ratio of the components of the elemental composition in this case can be explained by a denser structural packing observed when chromium cations are displaced from the structure of the crystal lattice, alongside a reduction in deformation stresses in the Mo–N crystalline bonds, which leads to an elevation in the stability of the crystal structure to external thermal effects resulting in oxidation. The formation of impurity inclusions in this case is due to the presence of local structural distortions in the crystal lattice, which leads to the formation of oxide phases, the growth of which occurs in the near-surface layer with subsequent evaporation from the surface or fixation near the grain boundaries due to desorption capacity. It is important to highlight that the compaction of the crystal structure leads to the creation of additional barrier obstacles to the diffusion of oxygen from the surface into the depths of the samples, which in turn leads to the inhibition of oxidation processes and the formation of oxide inclusions in the structure.

**Table 2:** Assessment of the weight contributions of the main and impurity phases in the composition of MoCrN coatings

Parameter	Sputtering power, W				
	200 W	220 W	250 W	270 W	300 W
Weight contribution of MoN, wt%	74.6	76.1	79.3	81.4	84.6
Weight contribution of MoO_3_, wt%	25.4	23.9	20.7	18.6	15.4

**Fig. 5:**
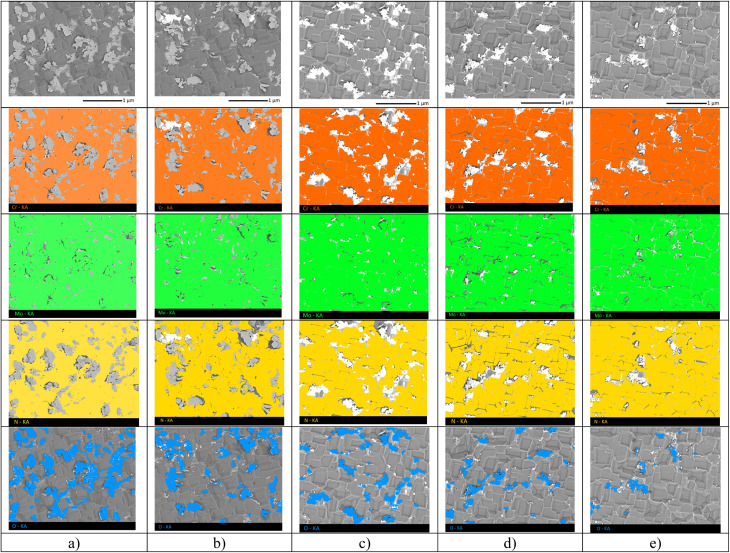
SEM images of the MoCrN coating surface after thermal testing: (a) 200 W; (b) 220 W; (c) 250 W; (d) 270 W; (e) 300 W.

The formation of inclusions in the form of MoO_3_, according to the X-ray phase analysis data, occurs in the form of finely dispersed inclusions with a distorted structure, which are in a metastable state. At the same time, the tendency of molybdenum oxide to desorption leads to the fixation of the impurity phase in the form of inclusions at the grain boundary. Direct confirmation of this phenomenon are the images of the coating surface shown in Fig. 5, which clearly show the presence of impurity inclusions on the coating surface after thermal testing. Analysis of the elemental composition of these inclusions showed that they consist of molybdenum and oxygen, which is a direct confirmation that these inclusions represent the oxide phase MoO_3_, the presence of which was determined using the X-ray phase analysis method. At the same time, it is evident that a change in the ratio of the component elements in the composition of the coatings leads to an increase in the resistance of the coatings to oxidation, which is manifested in a smaller number of formations on the surface of the coatings, alongside a decrease in the size of the impurity inclusions themselves in the composition of the coatings, which are mostly located near the grain boundaries in the composition of the coatings.

Tables 3 and 4 present the results of the assessment of changes in the hardness of the coatings, as well as the adhesive strength, which reflects the resistance of the coatings to detachment from the substrate surface in the case of the original samples, as well as after tribological tests at the wear site and after thermal tests. According to the presented data, changing the ratio of components in the coating composition with an increase in the proportion of molybdenum leads to an increase in the values of hardness and adhesive strength, from which it follows that there is a positive effect of varying the production conditions on the general mechanical properties of the coatings, and not only on changing wear resistance. In this case, the growth in hardness and adhesive strength values can be explained by the effects of removal of deformation structural distortions in the crystal lattice due to the substitution of chromium by molybdenum in the cubic lattice of MoN, as well as its compaction, which results in increased stability and increased resistance to external influences.

**Table 3:** Data on the evaluation of the hardness of the studied MoCrN coatings as a result of the study of resistance to wear and thermal effects

Experimental conditions	Hardness, GPa				
	Sputtering conditions				
	200 W	220 W	250 W	270 W	300 W
Initial values	1.63 ± 0.08	1.78 ± 0.06	2.21 ± 0.07	2.54 ± 0.06	2.78 ± 0.03
After standard measurements of tribological characteristics	1.52 ± 0.03	1.69 ± 0.04	2.11 ± 0.07	2.46 ± 0.05	2.73 ± 0.04
After thermo-tribological tests	1.45 ± 0.06	1.62 ± 0.03	2.03 ± 0.03	2.41 ± 0.04	2.68 ± 0.05
After tribo-corrosion tests	1.25 ± 0.07	1.50 ± 0.05	1.94 ± 0.06	2.25 ± 0.05	2.53 ± 0.03
After thermal corrosion tests	1.37 ± 0.04	1.58 ± 0.04	2.02 ± 0.05	2.36 ± 0.04	2.64 ± 0.06

**Table 4:** Data on the assessment of the adhesion strength of MoCrN coatings as a result of the study of resistance to wear and thermal effects

Experimental conditions	Adhesive strength (Lc3) (the amount of force applied to detach from the surface), N				
	Sputtering conditions				
	200 W	220 W	250 W	270 W	300 W
Initial values	84 ± 2	93 ± 3	105 ± 3	127 ± 3	144 ± 2
After standard measurements of tribological characteristics	78 ± 3	88 ± 3	100 ± 3	123 ± 2	141 ± 3
After thermo-tribological tests	74 ± 3	84 ± 4	96 ± 2	120 ± 3	138 ± 2
After tribo-corrosion tests	64 ± 2	78 ± 2	92 ± 3	112 ± 2	131 ± 3
After thermal corrosion tests	71 ± 3	82 ± 3	96 ± 4	117 ± 3	136 ± 4

During assessment of the strength characteristics of the coatings under study subjected to tribological tests, as well as thermal impact, indentation and scratch tests were performed from the area of damage associated with the impact of the counterbody during friction. The results presented in the tables reflect changes in strength properties associated with degradation as a result of external impacts. The general observed trend of decreases in strength parameters in the case of variation in the ratio of elements with variation in synthesis conditions, in comparison with the initial values, indicates an increase in the resistance of coatings to weakening and degradation caused by prolonged mechanical impact, as well as an aggressive environment and thermal heating. Moreover, the smallest changes in hardness and adhesive strength are observed for samples subjected to the standard tribological testing procedure, which indicates that in the absence of additional external influences associated with exposure to aggressive environments or high temperatures, the observed changes in strength characteristics are quite low, which indicates the stability and high strength of the coatings. In the case of samples exposed to the simultaneous action of aggressive environments or heating and mechanical friction, the decrease in strength parameters (hardness and adhesive strength) at the points of contact between the counterbody and the surface is more pronounced than in the case of the standard scheme for conducting tribological testing experiments. Such differences indicate that the combination of external influences with friction leads to an acceleration of surface wear processes due to the acceleration of processes of deformation distortion of the crystalline structure of coatings at the points of contact, caused by the penetration of oxygen with the subsequent formation of oxide inclusions, an increase in the proportion of which leads to accelerated wear and loss of strength.

**Fig. 6:**
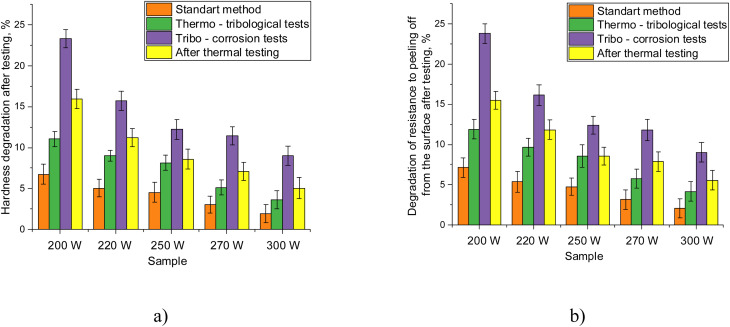
Results of the assessment of changes in strength parameters, calculated by comparing the values obtained after testing with the initial values of the coatings: (a) evaluation results of changes in hardness of MoCrN coatings, presented for different experiments in comparison with the initial values; (b) evaluation results of changes in adhesion strength for the studied MoCrN coatings, presented for different experiments in comparison with the initial values.

Based on the obtained data on changes in the hardness and adhesive strength of MoCrN coatings, presented in the tables, calculations were made to assess the degree of degradation of strength parameters depending on the type of experiments conducted. The data were obtained to compare the influence of different types of impacts on the stability of the strength properties of coatings, as well as to identify the role of alteration of the ratio of elements in the composition of coatings on resistance to degradation of strength characteristics. The calculation results are presented in Fig. 6.

A general analysis of the obtained dependencies of the change in hardness and adhesive strength presented in the figures makes it possible to conclude that alteration of the conditions of coating sputtering (power variation), leading to a change in the ratio of elements in the composition of the coatings (dominance of molybdenum) plays a key role in enhancement of resistance to external influences, including thermal heating of samples. According to the data presented, in the case of a standard test scheme, the assessment of changes in hardness and adhesive strength after completion of tests at the friction site showed a degradation of values of about 6–2.5%, depending on the ratio of elements in the composition. At the same time, the substitution of chromium by molybdenum leads to a more than twofold decrease in the degradation values of mechanical properties, which indicates a positive effect of variation in the elemental composition in coating samples.

According to the obtained results of assessment of the change in strength parameters reflecting the degree of degradation of hardness and adhesive strength under external influences, the greatest changes are observed in the case of tribo-corrosion tests, including the simultaneous impact of an aggressive environment and mechanical friction. In this case, the degradation degree varies from 23% for samples in which chromium predominates to 8–10% for samples in which molybdenum predominates. Moreover, by comparing the values of degradation values of two experiments (standard scheme and in the case of tribo-corrosion tests), one can conclude that, regardless of the ratio of elements in the composition of the coatings, the degree of degradation grows almost equally for all the samples studied. One of the explanations for such changes associated with more pronounced destruction and degradation of the surface in the case of tribo-corrosion tests may be the fact that under mechanical loads in the case of simultaneous exposure to an aggressive environment during friction in the near-surface layer, local metastable areas of delamination arise, which leads to a loss of strength, alongside destabilization of strength parameters, including adhesion to the substrate. At the same time, a change in the ratio of elements in the composition of MoCrN coatings, leading to an increase in the degree of structural ordering and compaction of the crystal lattice, leads to a reduction in the degree of degradation of strength properties, which has a positive effect on the properties of coatings and their resistance to external influences, as well as a slowdown in the destruction processes caused by both mechanical factors and external influences, including aggressive environments and high temperatures.

## Conclusion

The paper considers the possibility of enhancement of wear resistance of MoCrN coatings by variation of the ratio of components in the composition, the change of which was carried out by alteration of the magnetron sputtering power. At the same time, the studies are aimed not only at assessment of changes in the tribological characteristics of coatings depending on their elemental composition, but also at determination of the influence of additional external factors on changes in the wear resistance of coatings.

During the tribological tests of the studied MoCrN coatings, it was established that the combination of external influences associated with heating or exposure to an aggressive environment with mechanical friction leads to an acceleration of surface wear processes and a decrease in resistance to destruction under prolonged mechanical impact. It should be noted that variation in the elemental composition in MoCrN coatings leads to increased wear resistance, alongside reduction in the degradation rate when exposed to aggressive environments on the coating material.

During thermal tests it was established that the main effect of high-temperature heating obtained by contact of samples with an open flame leads to the formation of oxide inclusions in the near-surface layer, most of which are formed near the grain boundaries. At the same time, the compaction and removal of structural-deformation distortions in the crystal lattice of the MoN phase at substitution of chromium by molybdenum leads to an increase in the stability of the coatings to oxidation processes, which was clearly demonstrated using a comprehensive analysis of X-ray diffraction patterns and images of the surface morphology of the coatings.

The results obtained can be used in the future to predict and select the type of coatings to protect steel structures from the negative impact of external factors associated with extreme operating conditions, as well as to increase the wear resistance of materials exposed to constant external influences, including friction or mechanical loads.

## Conflicts of interest

The authors declare no conflicts of interest.

## Data Availability

All experimentally obtained data are presented in the article. All data presented in the article were obtained using certified equipment and proven analysis methods.
